# Transplant biopsy beyond light microscopy

**DOI:** 10.1186/s12882-015-0136-z

**Published:** 2015-08-07

**Authors:** Benjamin Adam, Michael Mengel

**Affiliations:** Department of Laboratory Medicine and Pathology, University of Alberta, T6G 2S2 Edmonton, Canada

**Keywords:** Allograft biopsy, Banff classification, Molecular pathology, Diagnostics

## Abstract

Despite its long-standing status as the diagnostic “gold standard”, the renal transplant biopsy is limited by a fundamental dependence on descriptive, empirically-derived consensus classification. The recent shift towards personalized medicine has resulted in an increased demand for precise, mechanism-based diagnoses, which is not fully met by the contemporary transplantation pathology standard of care. The expectation is that molecular techniques will provide novel pathogenetic insights that will allow for the identification of more accurate diagnostic, prognostic, and therapeutic targets. Here we review the current state of molecular renal transplantation pathology. Despite significant research activity and progress within the field, routine adoption of clinical molecular testing has not yet been achieved. The recent development of novel molecular platforms suitable for use with formalin-fixed paraffin-embedded tissue will offer potential solution for the major barriers to implementation. The recent incorporation of molecular diagnostic criteria into the 2013 Banff classification is a reflection of progress made and future directions in the area of molecular transplantation pathology. Transcripts related to endothelial injury and NK cell activation have consistently been shown to be associated with antibody-mediated rejection. Prospective multicenter validation and implementation of molecular diagnostics for major entities remains an unmet clinical need in transplantation. It is expected that an integrated system of transplantation pathology diagnosis comprising molecular, morphological, serological, and clinical variables will ultimately provide the greatest diagnostic precision.

## Review

### Introduction

Despite its limitations, the renal transplant biopsy still represents the diagnostic “gold standard” [1]. Its diagnostic precision is limited by the fact that assessment is mostly based on descriptive, empirically-derived consensus classifications that frequently lack an etiologic basis [2]. This approach is becoming increasingly insufficient in the era of personalized medicine, in which precise, mechanism-based diagnoses are the prerequisite for targeted treatment in the individual patient [3, 4]. The expectation is that molecular technologies will provide novel insights into disease mechanisms and thus allow for the identification of more accurate diagnostic, prognostic, and theranostic (i.e., predicting response to targeted treatment) biomarkers. Although significant progress and insights have been derived from molecular studies, widespread adoption of molecular diagnostics in renal transplantation pathology has not yet been accomplished. Further validation of specific applications leading to increased diagnostic precision in a clinically relevant setting is ongoing.

To date, the majority of the comprehensive data sets that include clinicopathological correlations have been derived from quantitative transcriptomics analysis of renal transplant biopsies and, to a lesser degree, urine and blood [5–11]. Despite this, urine provides a potentially unique, non-invasive window on the kidney, especially for those molecules that ‘leak’ into the urine in association with tubular injury [12–14]. The main focus for molecular diagnostic research in renal allografts was on rejection (T cell mediated and antibody-mediated) and the assessment of acute tissue injury, especially in the context of predicting organ function immediately post transplantation and late allograft failure [11, 15, 16]. Limited research data are available for assessing the molecular phenotype of relevant differential diagnosis like drug toxicity, in particular Calcineurin inhibitor nephrotoxicity, and viral or other infections in the allograft. Accordingly, we will focus here on reviewing the clinical value of integrating molecular assessments of rejection and acute allograft injury with standard histopathology assessment of renal allograft biopsies.

For most molecular platforms, extra biopsy material needs to be specifically procured and stabilized, potentially representing additional risk and inconvenience for the patient [17, 18]. However, high-throughput gene expression platforms that work with formalin-fixed, paraffin-embedded (FFPE) samples have recently become available. For example, the NanoString nCounter® Analysis System (Seattle, WA) is a molecular “barcode” probe-based technology that enables direct, digital quantification of nucleic acids without amplification, using low amounts of short fragments of total RNA, miRNA, or DNA [19]. The nCounter® system is highly multiplexed, with the capacity of quantifying up to 800 targets simultaneously, and has been shown to be more sensitive than microarrays and similar in sensitivity to real-time PCR [19, 20]. Platforms like this have the potential to enable translation of multi-parametric molecular signatures from omics discovery studies into clinical FFPE diagnostics using the same sample that is reviewed under the microscope. This would allow for practical integration of molecular diagnostics into the current standard of care. Furthermore, the ability to apply molecular quantification techniques to FFPE samples introduces the possibility of conducting large retrospective validation studies for new molecular diagnostics on well annotated samples with long-term follow-up.

### Beyond the light microscope: molecular assessment of donor biopsies

Organ donation and especially brain death induces a significant molecular response in donor tissue [21, 22]. Qualitative assessment of a donor organ, with the aim of guiding allocation and post-transplantation management to achieve the best possible function and outcome in the recipient, represents a major unmet clinical need. Numerous histological, clinical, and combined scoring and assessment systems for donor organs have been described, but a significant proportion of harvested donor kidneys is still discarded without robust evidence.

At the transcriptional level, the response to tissue injury presents as a reduction in the expression of genes related to cell transport and an up-regulation of cell cycle, repair and tissue remodelling transcripts along with embryonic pathways like *wnt* and *notch* [23, 24]. In addition, genes associated with immune, signal transduction, and oxidative stress responses have been shown to be increased in donor biopsies from recipients with impaired function post-transplantation [25]. The mRNA expression of well-known acute kidney injury (AKI) biomarkers like LCN2 and HAVCR1/KIM-1 has been found to be significantly increased in recipients developing delayed graft function (DGF) [26]. In microarray studies of time-zero biopsies taken post-reperfusion, unsupervised analysis separated donor kidneys into three groups: living donors, low-risk deceased donors, and high-risk deceased donors who demonstrated the greatest incidence of DGF [21]. The strongest correlate with early dysfunction in the high-risk donors was mean expression of a set of 30 injury transcripts. These included well-known biomarkers of AKI primarily expressed by tubular epithelial cells like osteopontin M receptor, integrin β6, lipocalin 2, versican, cathepsin S, and cadherin 6 [27]. Molecular assessment of donor biopsies therefore has the potential to quantify tissue injury and identify organs at risk for DGF. This can guide the development of specific post-transplant strategies for these high-risk donor organs and consequently reduce current discard rates.

### Beyond the light microscope: molecular diagnosis of T Cell-Mediated Rejection (TCMR)

Numerous studies have described the molecular phenotype of renal allograft biopsies presenting with the characteristic histological features of TCMR (i.e., interstitial infiltration and tubulitis): transcripts expressed by subsets of T lymphocytes (cytotoxic T lymphocytes (CTLs), effector memory T cells, T helper cells, regulatory T cells [28–31]), transcripts expressed by macrophages [32, 33], and transcripts regulated by interferon-gamma (IFNγ) [34, 35]. These include prototypical CTL transcripts like granzyme B, perforin, and Fas ligand [10, 18, 36–41]. Included among the numerous IFNγ-regulated cytokines and chemokines associated with acute rejection were TGFβ, TNFα, RANTES, MIP-1α, HLA class I and II molecules, CXCL9, CXCL10, and CXCL11 [10, 42, 43]. Genome-wide microarray discovery studies confirmed earlier hypothesis-based PCR studies and revealed that numerous transcripts show similar expression patterns under the same disease condition [5, 15, 43, 44]. Sarwal and colleagues were the first to apply genome-wide microarrays for systematic screening of genes expressed in rejecting grafts. This study confirmed the expression of T cell transcripts, but also found a B cell signature associated with subsequent graft failure [9].

Hundreds of individual members in the described sets of genes associated with TCMR change their expression in a highly correlated, stereotyped fashion, moving in large groups that reflect the major biological processes operating in renal allografts [11, 15, 43]. This observation was recently expanded upon in a comprehensive meta-analysis of human gene expression studies in allo graft rejection across all organ types [45]. The authors postulate an “Immunological Constant of Rejection” hypothesis based on the observation that different immune-mediated processes (i.e., allograft rejection, autoimmunity, infection, cancer, graft-versus-host disease, acute cardiovascular events, chronic-obstructive pulmonary diseases, and placental villitis) share common convergent final molecular mechanisms. Molecular features consistently described in these different immune- mediated tissue destruction processes include the activation of IFNγ-regulated genes, the recruitment of cytotoxic cells through massive production of respective chemokine ligands (primarily through CXCR3/CCR5 ligand pathways), and the activation of immune effector function genes (i.e., genes expressed by CD8-positive cytotoxic T cells and NK cells upon activation) [45].

Accordingly, the Edmonton group used their comprehensive collection of human renal allograft biopsy mRNA microarray data to build an unsupervised predictive TCMR classifier [42, 46]. This classifier summarizes the molecular groups of the fundamental immune-pathological mechanisms of rejection and assigns a percent probability score to each individual biopsy indicating the likelihood of TCMR based on its genome-wide molecular phenotype. Using such a classifier, molecular diagnostic thresholds for TCMR can be defined and potentially used for clinical decision making.

### Beyond the light microscope: molecular diagnosis of Antibody-Mediated Rejection (ABMR)

In 2013, the international Banff classification for allograft rejection underwent significant revision and now includes molecular assessment of antibody-mediated injury as a potential diagnostic criterion for ABMR independent of C4d staining [47]. Over the last several years, considerable evidence has emerged that previous Banff criteria for ABMR, due to its dependence on C4d, missed a significant proportion of ABMR cases. In parallel, underlying mechanisms of antibody-mediated tissue injury have become increasingly better understood [48].

In the vast majority of ABMR, anti-HLA antibodies find their target in the microcirculation of the allograft. This interaction between antibodies and the endothelial cell causes stress to the microcirculation, mediated through classical complement activation, direct interaction of the antibody with the endothelium, or cell-dependent cytotoxicity via Fc receptors on neutrophils, NK cells, or macrophages [49]. Such interactions between antibody and antigen lead to significant molecular changes in the endothelium of the kidney allograft microcirculation (i.e., peritubular capillaries and glomeruli). Sis et al. were the first to develop a literature-based set of transcripts with expression restricted to endothelial cells [50]. The authors found a significant up-regulation of endothelium-specific molecules, such as factor VIII / von Willebrand factor (VWF), melanoma cell adhesion molecule (MCAM/CD146), cadherin-5 (CDH5), selectin E (SELE/CD62E), platelet/endothelial cell adhesion molecule 1 (PECAM1/CD31), CD34, caveolin 1 (CAV1), in human renal allograft biopsies with histological features of antibody-mediated tissue injury and simultaneous presence of donor-specific antibodies (DSA).

Subsequently, as the correlate of antibody-facilitated cell-dependent cytotoxicity, molecules specific for NK cell infiltration were shown to be associated with microcirculation inflammation (i.e., capillaritis and glomerulitis) and the presence of DSA [51, 52]. By comparing the intragraft gene expression between patients with and without DSA, the following NK cell specific transcripts were found to be increased in biopsies from DSA-positive patients: fractalkine receptor (CX3CR1), myeloblastosis viral oncogene homolog-like 1 (MYBL1), fibroblast growth factor binding protein 2 (FGFBP2, also known as KSP37), killer cell lectin-like receptor F1 (KLRF1, also known as NKp80) and SH2 domain containing 1B (SH2D1B, also known as EAT2). In cases with ABMR pathology and the presence of DSA, expression of both endothelial and NK cell transcripts was independent of C4d staining results, with up to 60 % being C4d-negative.

Several groups have since independently confirmed these mechanistically derived associations between ABMR and endothelial, NK cell, and inflammatory transcripts. The Mayo group studied the intragraft gene expression profiles of positive crossmatch (+XM) kidney transplant recipients who do and do not develop transplant glomerulopathy (TG). Comparison of protocol renal allograft biopsies in + XM/TG+ and control groups revealed significantly altered expression in up to 3200 genes, including inflammatory, NK cell, and endothelial cell transcripts [53]. Similarly, colleagues from the Montefiore Medical Center, New York demonstrated differential intragraft gene expression profiles between patients with TG and graft loss and those with TG and functioning grafts. Endothelial transcripts, complement-associated transcripts, interleukins, and granulysin (CTL-associated) were significantly up-regulated in biopsies from the TG group with consecutive graft loss [54]. In addition, the same group demonstrated increased expression of gene transcripts related to CTLs, NK cells, macrophages, and IFNγ in both cases with DSA-positive/C4d-positive chronic ABMR and DSA-positive/C4d-negative TG, providing further evidence that DSA-positive TG represents true ABMR despite being negative for C4d [55, 56]. Independently, a microarray-based classifier was developed that assigned diagnostic ABMR probability scores to each biopsy. The transcripts distinguishing ABMR from other diagnoses were again mostly expressed in endothelial or NK cells or were IFNγ-inducible. The classifier scores correlated with the presence of microcirculation injury according to Banff lesion scores and DSA levels [57]. The fact that this classifier independently uses endothelial and NK cell-associated transcripts for diagnosing ABMR further corroborates the above described hypothesis-driven observations by others that such transcripts are specifically increased in renal allografts with ABMR.

These findings indicate that molecular studies, particularly with endothelial gene sets, can be used to measure ongoing ABMR activity leading to TG, which is associated with eventual progression to graft failure. Therefore, adding quantitative molecular assessments to the Banff classification for DSA-positive cases has the potential to increase diagnostic precision for C4d-negative antibody-mediated rejection.

### Beyond the light microscope: molecular prediction of allograft outcome

A molecular classifier for the prediction of allograft failure in clinically-indicated biopsies taken more than one year post-transplantation was described as being superior to all known clinical (creatinine, proteinuria, GFR) and morphological risk factors (Banff lesion scores and diagnoses) for allograft failure. Interestingly, the classifier utilized genes included in the acute molecular AKI response as the main source for prediction. This suggests that an ongoing active molecular injury response in the tissue, due to progressive diseases like rejection or glomerular diseases, is the main correlate for functional deterioration in late allograft biopsies [58]. Increased expression of acute injury response genes correlated with poor function and with inflammation in areas of fibrosis, but not with fibrosis without inflammation [59]. Many individual transcripts expressed in late biopsies from failing allografts were shared between the acute injury gene set [24, 59] and the molecular risk score derived from the predictive allograft survival classifier, including integrin β6 (ITGB6), versican (VCAN), and nicotinamide N-methyltransferase (NNMT). Therefore, at a molecular level, progression to failure is primarily a function of ongoing parenchymal injury caused by various specific diseases like recurring glomerulonephritis or ABMR.

## Conclusions

**Fig. 1:**
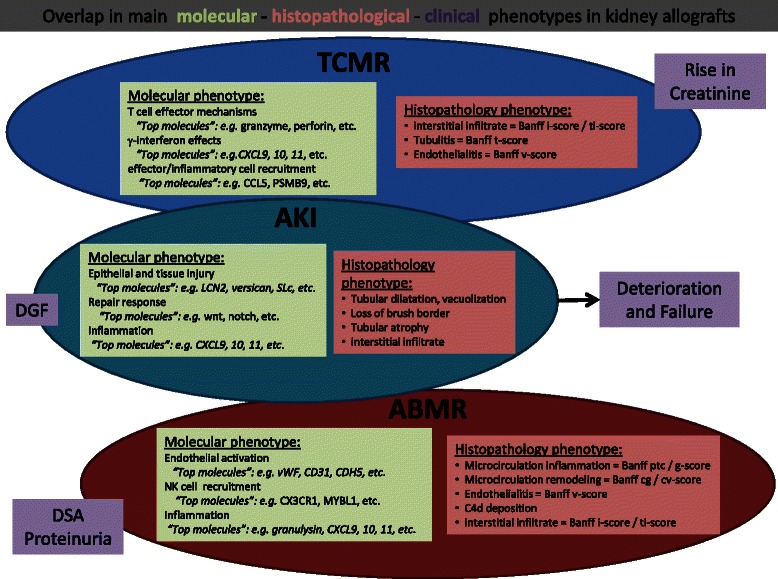
Overlap in main molecular - histopathological - clinical phenotypes in kidney allografts: The molecular phenotype in renal allografts presents as changes in groups / sets of genes/transcripts which are strongly correlated with each other. Few “top” genes can be selected to represent specific biological processes like T cell infiltration and activation. These molecular phenotypes correlate with related histological lesions and other serological parameters, e.g., microcirculation inflammation and DSA with increased expression of endothelial and NK cell genes in antibody-mediated rejection. Of note is that to some extent the different molecular phenotypes overlap between diagnostic entities and thus cannot be considered absolutely specific, i.e., similar to non-specific histological lesions

The results obtained from genome-wide expression studies in kidney transplant biopsies indicate that the vast majority of differentially expressed molecules represent a stereotyped tissue response to injury caused by and overlapping between different disease processes. However, within this non-specific injury and inflammation response, identification of patterns correlating with disease activity and prognosis can allow for disease-specific information to be elucidated (Fig. 1). Finding diagnostic specificity in large-scale molecular changes is as challenging as with non-specific morphological features like interstitial inflammation or tubular atrophy, which as well overlap between disease entities. It is unlikely that even the most comprehensive molecular assessments will provide absolute diagnostic precision. Although molecular assessment may be superior in some settings such as early tissue injury, which is essentially invisible to morphology, histopathology will always carry greater specificity and sensitivity in other entities like focal glomerular diseases. Therefore, a process for consensus generation will be needed to adopt molecular diagnostics (including related biostatistical algorithms) as it was the case for histopathological diagnostic criteria through the Banff process [2]. Given that relative small sets of genes can provide measurements of relevant aspects of different disease processes, consensus is needed in regard to which genes should be measured and which thresholds should be considered diagnostically and clinically relevant. But, deriving a diagnostic molecular signature and relevant diagnostic thresholds from large-scale, high-dimensional omics data is a major challenge in the absence of a true diagnostic gold standard for training and validation purposes. This is the case with transplantation pathology in that the Banff classification system is empirically-derived and consensus-based but not necessarily representative of true pathogenetic and biological disease mechanisms [2].

It is therefore likely that an integrated diagnostic system comprising molecular, morphological, serological, and clinical variables, as proposed in the revised 2013 Banff classification, will provide the greatest diagnostic precision [47]. The adoption of such a refined, integrated diagnostic system into clinical practice will require ongoing interdisciplinary multicenter efforts for conducting well-designed validation studies. With the recent availability of novel molecular technologies and user-friendly biostatistics tools, these studies can now be conducted both retrospectively and prospectively. This provides the opportunity for robust outcome-based validation of molecular diagnostics independent of current arbitrary histology-based diagnostic consensus.

## Abbreviations

ABMR: Antibody-mediated rejection
AKI: Acute kidney injury
CTL: Cytotoxic T lymphocyte
DGF: Delayed graft function
DSA: Donor specific antibody
FFPE: Formalin-fixed paraffin embedded
TG: Transplant glomerulopathy
TCMR: T cell mediated rejection
XM: Crossmatch
